# Computational analysis of non-synonymous SNPs in the sheep MC4R gene

**DOI:** 10.1016/j.jgeb.2026.100709

**Published:** 2026-05-15

**Authors:** Anila Hoda, Sulltane Ajçe, Ilia Mikerezi, Xhelil Koleci

**Affiliations:** a Academy of Sciences of Albania, Sheshi “Fan Noli”, Nr 7, Tirana, Albania; b University Fan S. Noli, Koça. Albania; c Agricultural University of Tirana, Albania, Rruga Paisi Vojdica, Kodër Kamëz, Tirana, Albania

**Keywords:** Protein stability, Conservation analysis, Protein structure, Molecular modeling, Computational biology, Functional genomics

## Abstract

The melanocortin-4 receptor (MC4R) gene is central to appetite regulation, body weight, and energy balance in vertebrates, with significant influence on sheep growth, fat deposition, feed efficiency, and reproduction. However, the functional consequences of non-synonymous single nucleotide polymorphisms (nsSNPs) in this gene remain unclear. This study aimed to evaluate the structural and functional impact of non-synonymous single nucleotide polymorphisms (nsSNPs) in the ovine MC4R gene using an integrated computational approach. A set of nsSNPs was analyzed to identify potentially deleterious variants based on their predicted effects on protein function, stability, and evolutionary conservation. A total of 12 ovine MC4R nsSNPs were identified from Ensembl and UniPro , analyzed using an integrated computational framework.. Functional effects were predicted by SIFT, PolyPhen-2, SNPs&GO, and PhD-SNP, while protein stability was assessed with I-Mutant, MUpro, CUPSAT, and DynaMut. Conservation analysis (ConSurf), structural modeling (SOPMA, I-TASSER, GalaxyWEB), validation (PROCHECK, ProSA), and molecular dynamics simulations (100 ns, WEBGRO) were performed. Four nsSNPs (R220G, G98R, E100K, Y187C) were consistently predicted as deleterious, and damaging nsSNPs often occurred in conserved regions. . Structural modeling and molecular dynamics simulations revealed that these nsSNPs may alter protein stability, flexibility, and conformational dynamics compared to the wild-type protein.. These findings identify several nsSNPs as potential candidate variants that may influence MC4R structure and function. However, their biological and practical relevance should be interpreted with caution, as the results are based solely on computational predictions. The identified variants represent promising targets for further investigation, but their application in selective breeding programs requires validation through population-based studies, genotype–phenotype association analyses, and experimental approaches. Overall, this study provides a framework for prioritizing potentially functional MC4R variants, rather than direct evidence for their use in breeding applications.

## 1 Introduction

The melanocortin-4 receptor (*MC4R*) gene encodes a G-protein-coupled receptor that plays a key role in regulating energy balance, appetite, and body weight in vertebrates ^1^. In livestock, especially sheep, genetic variations in *MC4R* have been linked to important economic traits. These include growth rate, meat quality, fat deposition, reproductive hormone levels, and feed efficiency 2, 3, 4, 5, 6. Among these genetic variations, non-synonymous single nucleotide polymorphisms (nsSNPs) are of particular interest because they alter the amino acid sequence of proteins and may affect their structure and function. These nsSNPs cause changes in amino acid sequences, which can directly affect protein structure and function ^7^. nsSNPs may influence protein stability or alter interactions with other molecules. Some nsSNPs may be harmless, while others may have strong effects on phenotype. In animal genetics, they are useful as molecular markers for selecting animals with desirable traits.

The importance of studying nsSNPs in livestock has grown, especially for improving breeding programs. They represent a key source of genetic variability. Marker-assisted selection can benefit from identifying functionally relevant nsSNPs ^8^. While MC4R has been extensively studied in pigs and cattle, its structural and functional variation in sheep remains poorly explored. With the growing availability of genomic resources, computational tools now allow fast and cost-effective prediction of nsSNP impact on protein function and structure 9, 10.

In contrast, ^11^ reported a regulatory variant (g.1016 G > A) located in the 3′UTR of MC4R in Hu sheep. This mutation was associated with birth and weaning weights but does not result in an amino acid change. While these studies show the potential significance of MC4R polymorphisms, most of them have focused on phenotypic traits. There is still a lack of detailed structural and functional analyses using *in silico* methods.

Studying MC4R snSNPs in sheep can improve our understanding of how genetic changes influence traits related to productivity. This knowledge supports the development of precise genetic tools for breeding. Since functional annotations in sheep are limited compared to model organisms, computational methods offer a powerful way to predict gene function. They also enable cross-species comparisons and structure-based predictions ^12^.

Although numerous *in silico* studies have investigated the functional impact of nsSNPs in various genes and species, comprehensive analyses focusing on the ovine MC4R gene remain limited. Most previous studies on MC4R in sheep have primarily explored associations between genetic polymorphisms and phenotypic traits, with little emphasis on the structural and functional consequences of these nsSNPS at the protein level. In this context, the present study provides a novel integrative approach by combining multiple computational tools to evaluate the impact of nsSNPs on protein function, stability, evolutionary conservation, and structural dynamics. By incorporating molecular modeling and molecular dynamics simulations, this work offers deeper mechanistic insights into how specific amino acid substitutions may affect MC4R function. The identification of deleterious nsSNPs contributes to the development of potential molecular markers for improving economically important traits in sheep breeding programs. Non-synonymous single nucleotide polymorphisms (nsSNPs), which result in amino acid substitutions, were the focus of this study.

Therefore, this study aims to perform a comprehensive *in silico* characterization of nsSNPs in the ovine MC4R gene, integrating functional prediction, structural modeling, and molecular dynamics simulations to identify key nsSNPs with potential biological and breeding significance..

## 2 Material and methods

### 2.1 Collecting SNPs and protein sequence from databases

Single nucleotide polymorphisms (SNPs) of the sheep MC4R (Melanocortin 4 Receptor) gene were collected from the Ensembl Genome Browser (https://ensembl.org/) by selecting “Sheep” as the species and searching with the gene symbol MC4R. The transcript sequence (Ensembl Transcript ID: ENSOART00020156170.1) and the corresponding gene ID (ENSOARG00020072061.1) were retrieved from the Ensembl database. Additionally, the protein sequence of the MC4R gene was obtained from the same source. All available variants associated with the MC4R gene were exported from the Ensembl Variant Table in CSV format. A total of 593 variants were initially identified. These variants were subsequently filtered in a stepwise manner. First, only variants located within the coding region were retained. Second, variants were classified according to their functional consequence based on Ensembl annotation. Only non-synonymous single nucleotide polymorphisms (nsSNPs), corresponding to missense variants that result in amino acid substitutions, were selected for further analysis. Synonymous variants, intronic variants, and variants located in untranslated regions (UTRs) were excluded from the analysis.

The UniProt ID for the MC4R protein (UniProtKB: B2MV59) was retrieved from the UniProt Protein Database (https://www.uniprot.org/), providing reference for further structural and functional analysis.

### 2.2 Functional prediction of nonsynonymous SNPs in the MC4R gene

To assess the potential functional impact of nonsynonymous single nucleotide polymorphisms (nsSNPs) in the MC4R gene, four online predictive tools were employed, each utilizing distinct algorithms. The primary protein sequence of MC4R was used as input for all analyses. The SIFT tool (https://sift.jcvi.org/) was used to evaluate whether amino acid substitutions are likely to affect protein function, based on sequence homology and the degree of conservation at each position. SNAP2 (https://rostlab.org/services/snap2web/) applies a neural network-based machine learning approach to classify variants as either “effect” or “neutral,” considering various sequence features. SNPs&GO (https://snps.biofold.org/snps-and-go/) integrates Gene Ontology (GO) terms with sequence information to predict disease-related nsSNPs. Finally, PhD-SNP (https://snps.biofold.org/phd-snp) was used to estimate the likelihood of a mutation being pathogenic, based on support vector machine (SVM) learning models trained on known disease-related variants. Together, these tools provide a comprehensive prediction of the functional consequences of nsSNPs in the MC4R protein.

### 2.3 Assessment of amino acid substitutions and their impact on protein stability

To evaluate the effects of amino acid substitutions on the structural stability of the MC4R protein, several computational tools were employed. CUPSAT (https://cupsat.tu-bs.de/) ^13^ was used to assess the impact of point nsSNPs based on structural and torsional potentials, analyzing the conformational flexibility of residues and atom-level interactions. The influence of nsSNPs on protein stability and dynamics was further investigated using DynaMut (https://biosig.unimelb.edu.au/dynamut/) ^14^, which integrates normal mode analysis, graph-based signatures, and vibrational entropy calculations to estimate changes in flexibility and Gibbs free energy (ΔΔG) upon mutation.

Additionally, I-Mutant 2.0^15^, a support vector machine (SVM)-based web server, was used to predict the effect of single-point nsSNPs on protein stability by estimating ΔΔG values in kcal/mol. A negative ΔΔG indicates decreased stability, while a positive value suggests enhanced stability. MUpro ^16^, which combines SVM and neural network algorithms, was also applied to predict the direction and confidence of stability changes. MUpro provides a score between –1 and + 1, where negative values predict destabilization, positive values indicate stabilization, and the magnitude reflects the prediction confidence. Together, these tools offer a comprehensive *in silico* framework for assessing how specific amino acid substitutions may alter the thermodynamic stability of the MC4R protein.

### 2.4 Sequence conservation analysis of MC4R variants using ConSurf

Sequence conservation of the MC4R protein was evaluated using the ConSurf web server (https://consurf.tau.ac.il), which estimates the evolutionary conservation of amino acid residues based on phylogenetic relationships and structural context. Each residue is assigned a conservation score ranging from 1 to 9, where 1–3 indicate highly variable residues, 4–6 denote moderately conserved positions, and 7–9 represent conserved or highly conserved sites ^17^. Residues with high conservation scores are often critical for maintaining structural integrity or functional activity. Therefore, amino acid substitutions occurring in highly conserved regions were considered potentially deleterious, as they may interfere with essential structural or functional roles within the MC4R protein.

### 2.5 Prediction of secondary structure of the MC4R protein

The secondary structure of the sheep MC4R protein was predicted using the Self-Optimized Prediction Method with Alignment (SOPMA), a computational tool that forecasts secondary structural elements based on multiple sequence alignment and statistical algorithms ^18^. SOPMA identifies common structural motifs including α-helices, β-sheets, β-turns, and random coils.

### 2.6 Tertiary structure modeling, refinement, and validation of the MC4R protein

The initial tertiary structure of MC4R was generated using the I-TASSER server (https://seq2fun.dcmb.med.umich.edu//I-TASSER/) ^19^, which constructs models through iterative threading and fragment assembly simulations. The model with the highest C-score, indicating the best confidence estimate, was selected for refinement.

To enhance the structural accuracy of the model, refinement was carried out using the GalaxyWEB server (https://galaxy.seoklab.org/index.html), which improves local geometry in loop and terminal regions and applies molecular dynamics-based relaxation for global structural optimization ^20^.

The final refined model was validated using two widely accepted structural assessment tools. PROCHECK (https://saves.mbi.ucla.edu/) ^21^ was used to evaluate stereochemical quality through Ramachandran plot analysis, while ProSA-web (https://prosa.services.came.sbg.ac.at/prosa.php) ^22^ assessed the overall energy profile and compared it to known native protein structures.

### 2.7 Prediction of Protein–protein interactions

Protein–protein interaction (PPI) analysis was performed using the STRING database (https://string-db.org/) ^23^, a comprehensive resource for exploring known and predicted protein associations. The amino acid sequence of MC4R was submitted as the input query to identify potential interaction partners based on experimental data, computational predictions, and existing literature.

### 2.8 Mutant generation and interaction analysis

The UCSF Chimera software ^24^ was used to model the mutations. Amino acid substitutions were introduced using the “Rotamers” tool to ensure accurate side-chain conformations. The resulting mutant structures were energy-minimized and prepared for further computational analysis.

### 2.9 Molecular dynamics simulations

To investigate the structural impact of nsSNPs in MC4R, all-atom molecular dynamics (MD) simulations were performed using the online WEBGRO Protein in Water Simulation platform (https://simlab.uams.edu/ProteinInWater/index.html). The simulations employed the GROMOS96 43a1 force field along with the SPC water model, using a triclinic box system with sodium chloride to mimic physiological conditions. Energy minimization was carried out using the steepest descent algorithm with 5000 steps. System equilibration was performed under both constant volume (NVT) and constant pressure (NPT) conditions at 300 K and 1 bar. The Leap-frog integrator was used for a 100-nanosecond production run, with a total of 1000 frames generated for each simulation 25, 26, 27.

Key structural parameters were analyzed to assess conformational dynamics and stability, including root-mean-square deviation (RMSD), root-mean-square fluctuation (RMSF), radius of gyration (Rg), solvent-accessible surface area (SASA), and hydrogen bond formation. These parameters were calculated using GROMACS tools (rms, rmsf, gyrate, sasa, and hbond), and the resulting data were visualized using Grace software (https://plasma-gate.weizmann.ac.il/Grace/).

The nsSNPs selected for molecular dynamics simulations (R220G, G98R, E100K, and Y187C) were chosen based on their consistent classification as deleterious across multiple functional prediction tools and their predicted impact on protein stability and evolutionary conservation. Variants showing strong destabilizing effects and/or localization in conserved regions were prioritized, as they are more likely to induce significant structural and functional alterations.

This selection strategy ensured that MD simulations focused on biologically relevant variants with the highest predicted functional impact.

## 3 Results

### 3.1 *In silico* functional prediction of MC4R variants

From the initial pool of 593 MC4R variants retrieved from Ensembl, twelve nsSNPs met the selection criteria and were subjected to detailed *in silico* analysis. The functional impact of nsSNPs induced amino acid substitutions in the sheep MC4R protein was evaluated using four widely recognized *in silico* tools: PolyPhen-2, SNPs&GO, SIFT, and PhD-SNP. Although these tools utilize different algorithms—ranging from sequence conservation and structural features to machine learning approaches—they provided consistent and reliable predictions across most variants. The results are summarized in the accompanying Table 1. A consensus-based classification was applied to improve the reliability of variant interpretation across different prediction tools. Variants such as G233S (rs1094063123) and T112M (rs3504151427) were consistently classified as tolerated or neutral by SIFT, SNPs&GO, and PhD-SNP, with low probabilities of pathogenicity. Similarly, V228L (rs3504425907) and M79T (rs3505205758) were predicted to be benign or neutral across multiple platforms, suggesting limited or no impact on protein function.

**Table 1:** Functional prediction of sheep MC4R nsSNPs using multiple *In Silico* tools.

No.	rsID	AA Change	SIFT (Score)	SIFT Class	PhD-SNP (Prob.)	PhD-SNP Class	PANTHER (Prob.)	PANTHER Class	SNPs&GO (Prob.)	SNPs&GO Class	Final Prediction
1	rs1093301379	A259V	0.02	Deleterious	0.659	Disease	0.449	Neutral	0.564	Disease	Mixed
2	rs1090795282	E100K	0.01	Deleterious	0.599	Disease	0.850	Disease	0.747	Disease	Deleterious
3	rs1094063123	G233S	0.52	Tolerated	0.511	Disease	0.250	Neutral	0.240	Neutral	Neutral
4	rs404529322	G98R	0.00	Deleterious	0.616	Disease	0.488	Neutral	0.506	Disease	Deleterious
5	rs3503148901	I173M	0.00	Deleterious	0.374	Neutral	0.716	Disease	0.569	Disease	Mixed
6	rs3505205758	M79T	0.02	Deleterious	0.334	Neutral	0.768	Disease	0.488	Neutral	Mixed
7	rs3505205771	N294S	0.02	Deleterious	0.889	Disease	0.683	Disease	0.721	Disease	Deleterious
8	rs3503686941	Q307E	0.03	Deleterious	0.502	Disease	0.609	Disease	0.601	Disease	Deleterious
9	rs596678149	R220G	0.00	Deleterious	0.901	Disease	0.898	Disease	0.826	Disease	Deleterious
10	rs3504151427	T112M	0.07	Tolerated	0.200	Neutral	0.339	Neutral	0.109	Neutral	Neutral
11	rs3504425907	V228L	0.00	Deleterious	0.237	Neutral	0.228	Neutral	0.216	Neutral	Neutral
12	rs1087446456	Y187C	0.01	Deleterious	0.809	Disease	0.935	Disease	0.759	Disease	Deleterious

SIFT scores < 0.05 indicate deleterious variants. Predictions from PhD-SNP, PANTHER, and SNPs&GO are classified as “Disease” or “Neutral” based on probability scores. Final prediction was assigned based on consensus among tools.

In contrast, several nsSNPs were identified as potentially pathogenic. Notably, R220G (rs596678149) and Y187C (rs1087446456) were consistently predicted to be deleterious or disease-causing, with high reliability indices and strong probability scores across SIFT, PhD-SNP, SNPs&GO, and PolyPhen-2. Additionally, E100K (rs1090795282) and G98R (rs404529322) were classified as deleterious by SIFT and disease-causing by the majority of other tools, reinforcing their likely pathogenic effects. These findings highlight several variants of interest for further experimental validation and potential association with functional or phenotypic changes.

### 3.2 *In silico* stability analysis of sheep MC4R variants using multiple prediction tools

The structural stability of the sheep MC4R protein upon twelve amino acid substitutions was assessed using four computational tools: I-Mutant, MUpro, CUPSAT, and DynaMut (Table 2). These tools predict the thermodynamic and dynamic consequences of point mutations. Most variants, including R220G, M79T, and N294S, consistently showed negative ΔΔG values across multiple tools, suggesting a strong destabilizing effect on MC4R protein structure.

**Table 2:** Predicted stability impact of MC4R variants using multiple estimation tools.

No.	Variant	mCSM (ΔΔG)	SDM (ΔΔG)	DUET (ΔΔG)	ENCoM (ΔΔG)	DynaMut (ΔΔG)	Stability Trend
1	A259V	+0.54	−0.61	+0.83	+0.38	+1.22	Mixed
2	E100K	−0.37	−1.22	−0.24	+0.45	+0.76	Destabilizing
3	G233S	−0.68	−0.95	−0.51	+0.00	+0.09	Destabilizing
4	G98R	−0.83	−2.59	−0.89	+0.80	+0.16	Destabilizing
5	I173M	−0.64	−0.43	−0.52	+0.03	+0.62	Destabilizing
6	M79T	−1.97	−0.86	−1.65	−0.57	−0.98	Strongly destabilizing
7	N294S	−1.33	−1.66	−1.45	−0.18	−0.23	Strongly destabilizing
8	Q307E	−0.17	+1.14	+0.54	−0.02	+0.25	Mixed
9	R220G	−0.93	−0.85	−0.96	−0.42	−1.00	Strongly destabilizing
10	T112M	+0.26	+0.63	+0.43	+0.07	+0.14	Stabilizing
11	V228L	−0.28	+0.39	+0.22	−0.11	+0.08	Mixed
12	Y187C	−0.32	+0.10	−0.11	−0.22	−0.06	Destabilizing

ΔΔG values are expressed in kcal/mol. Negative values indicate destabilizing effects, while positive values indicate stabilizing effects. The overall stability trend was determined based on the consensus of multiple prediction tools.

According to I-Mutant, ten out of twelve variants exhibited negative ΔΔG values, indicating decreased protein stability. Notably, variants G233S, R220G, G98R, M79T, I173M, and E100K had strongly negative values (< –1.0 kcal/mol), suggesting a substantial destabilizing effect. Conversely, Q307E (0.32 kcal/mol), A259V (0.41 kcal/mol), and Y187C (0.72 kcal/mol) were predicted to enhance stability.

MUpro provided more conservative predictions, classifying all twelve variants as destabilizing. Among these, R220G (–2.123), M79T (–1.489), and Y187C (–1.106) were the most destabilizing, indicating a high likelihood of structural disruption.

CUPSAT identified ten variants as destabilizing. The most destabilizing substitutions were M79T (–2.82 kcal/mol) and G98R (–1.46 kcal/mol). Only V228L (0.66 kcal/mol) and Y187C (0.46 kcal/mol) were classified as stabilizing.

**Fig. 1:**
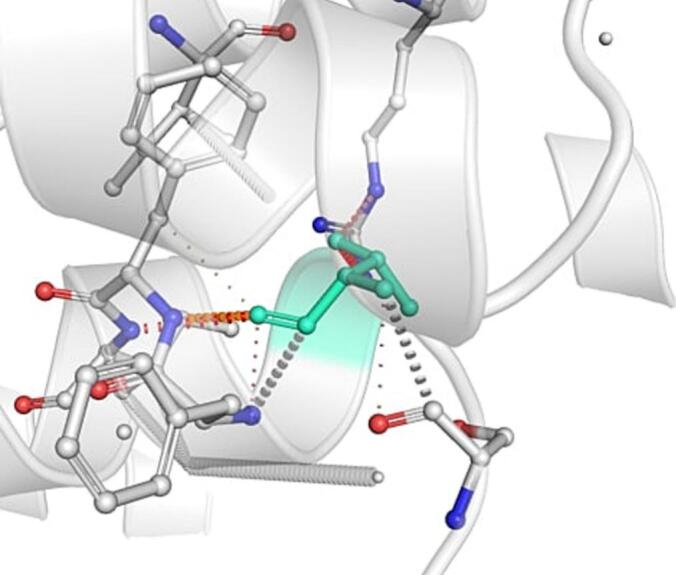

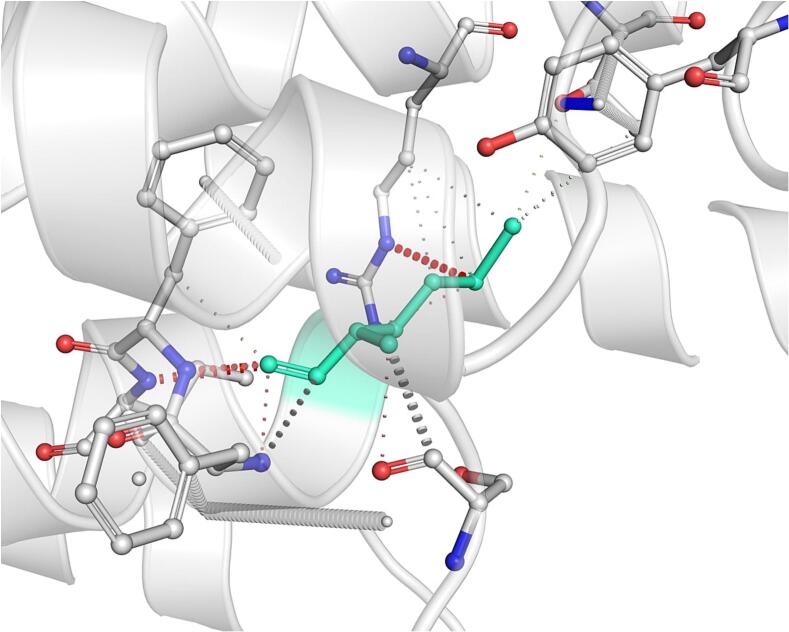

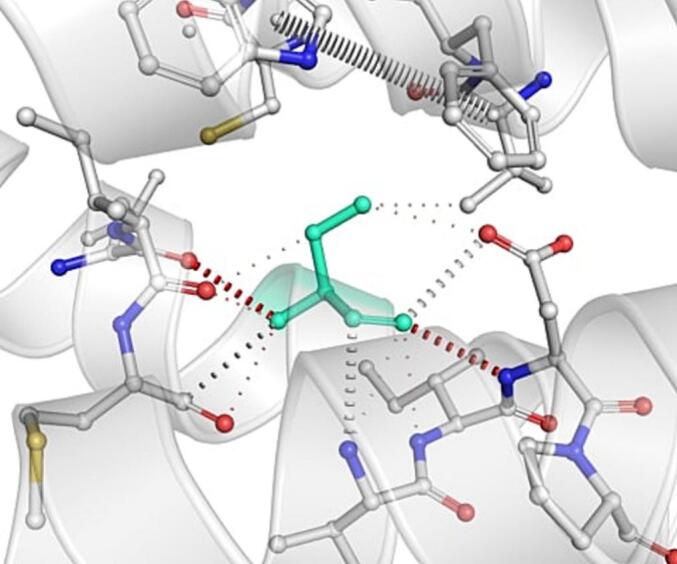

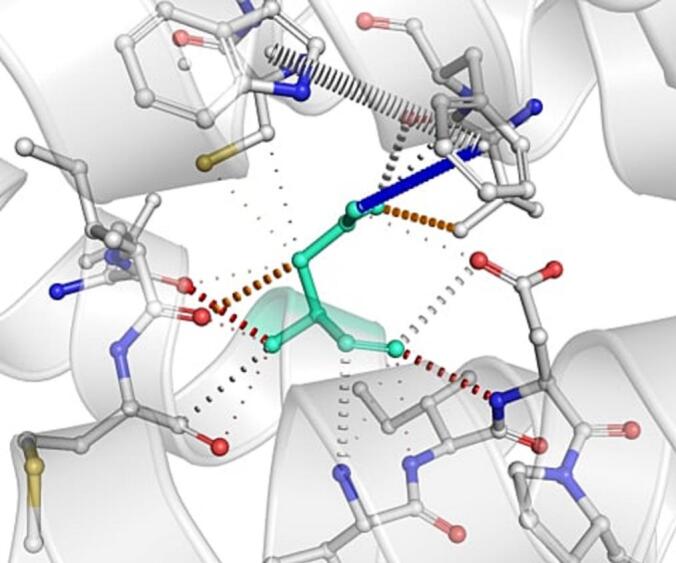

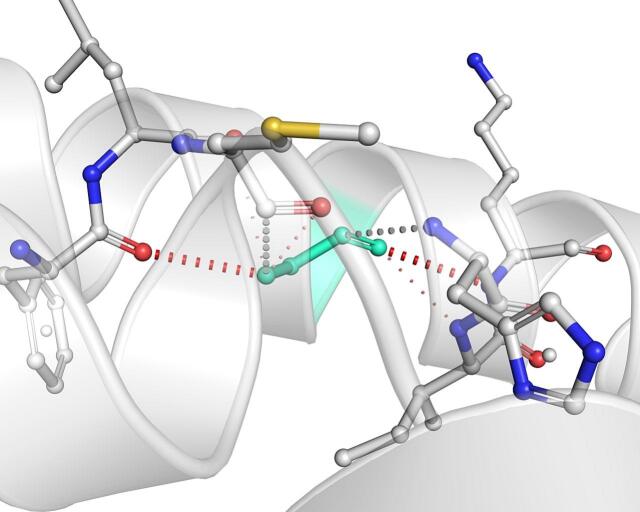

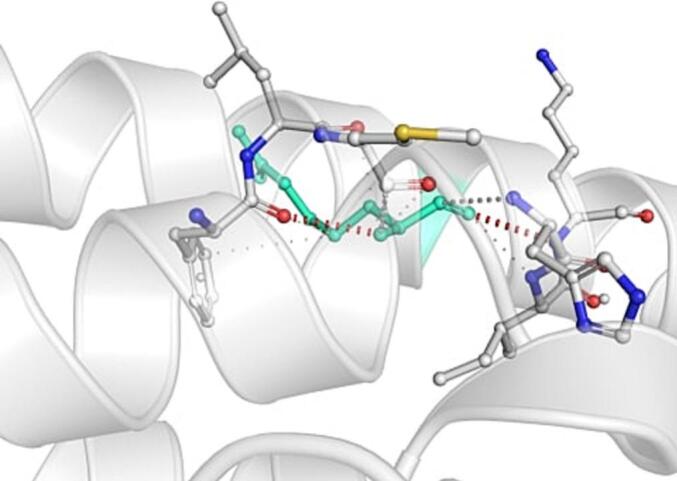

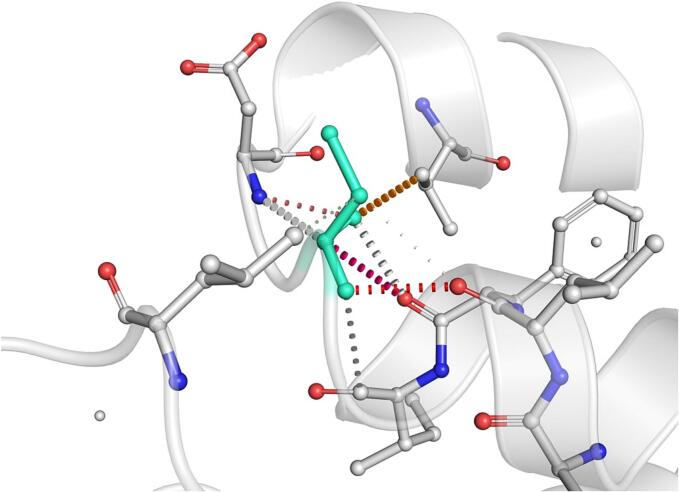

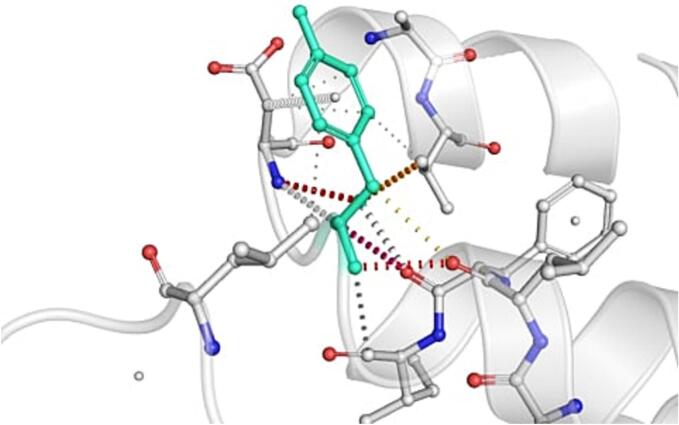
Structural impacts of the amino acid substitutions on MC4R protein stability computed by DynaMut (a) M79T; (b) M79T wild-type; (c) N294S; (d) N294S wild-type; (e) R220G; (f) R220G wild-type; (g) Y187C; (h) Y187C wild-type. NsSNPs are shown with altered hydrogen bonding and non-covalent interactions, indicating potential changes in local structural stability. Variants predicted to be destabilizing exhibit reduced or altered interaction networks compared to the wild-type structure.

DynaMut further analyzed the effect of these nsSNPs on both stability and flexibility by estimating changes in Gibbs free energy (ΔΔG) and vibrational entropy (ΔΔS_vib). nsSNPs such as M79T (–0.977 kcal/mol), R220G (–1.000 kcal/mol), and N294S (–0.228 kcal/mol) were predicted to be destabilizing, with increased flexibility that may weaken local structural integrity. In contrast, A259V, I173M, and E100K showed positive ΔΔG values (up to + 1.219 kcal/mol), suggesting a stabilizing effect. T112M and Q307E had mild stabilizing effects, while Y187C, G233S, V228L, and G98R were classified as having a neutral or moderate impact. Taken together, variants such as R220G, G98R, M79T, E100K, and I173M were consistently predicted to destabilize the MC4R protein across multiple tools, suggesting their potential to impair structural integrity and function. The differences in interatomic interactions, including hydrogen bonds and ionic interactions, between the wild-type and destabilizing mutant variants are illustrated in Fig. 1.

### 3.3 Conservation assessment of nsSNP impact using ConSurf analysis

**Fig. 2:**
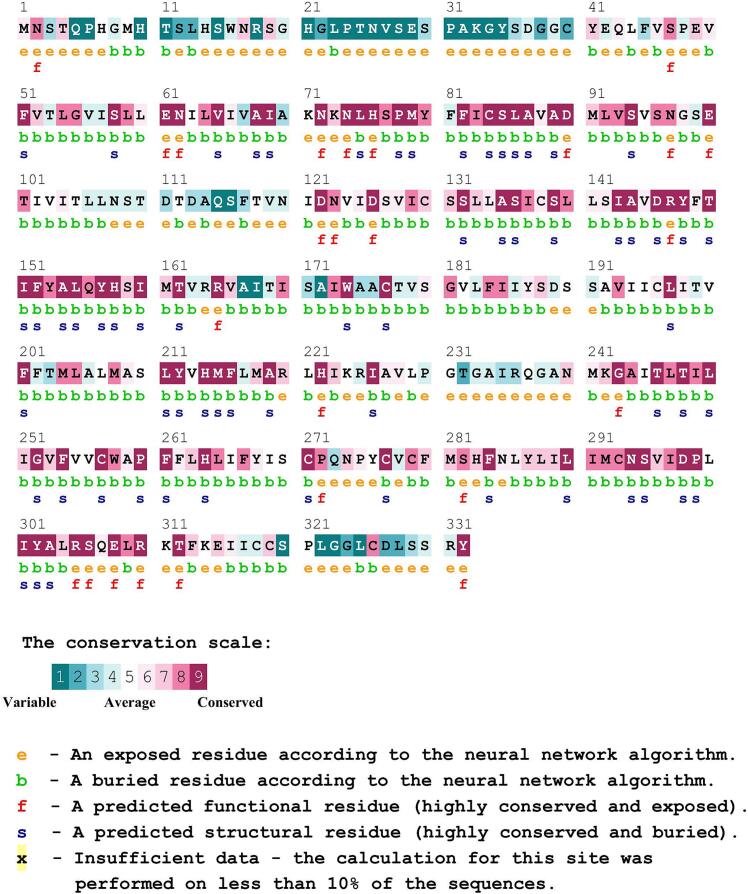
Residue conservation and functional annotation of sheep MC4R predicted by ConSurf. Amino acid residues are colored according to their evolutionary conservation scores (1–9), where highly conserved residues are shown in dark colors and variable residues in lighter colors. Residues predicted to be functionally important (highly conserved and exposed) and structurally important (highly conserved and buried) are indicated. The positions of analyzed nsSNPs are highlighted to show their distribution across conserved and variable regions.

To assess residue-level evolutionary conservation within the MC4R protein, the ConSurf web server was used, which applies a Bayesian algorithm to estimate conservation scores for each amino acid based on phylogenetic relationships and structural context. Twelve nonsynonymous single nucleotide polymorphisms (nsSNPs) were mapped onto the conservation profile generated by ConSurf (Fig. 2). Among these, six variants (A259V, V228L, Y187C, I173M, G98R, and M79T) were located in highly conserved and buried regions, suggesting that these residues contribute to the protein’s structural stability. One variant, Q307E, was located in a highly conserved and exposed region, indicating a likely functional role and the potential to affect protein activity or molecular interactions. In contrast, variants such as G233S, T112M, R220G, and E100K were found in moderately conserved or variable regions, with no strong structural or functional annotations, suggesting a milder or context-dependent effect.

### 3.4 Secondary structure prediction

**Fig. 3:**
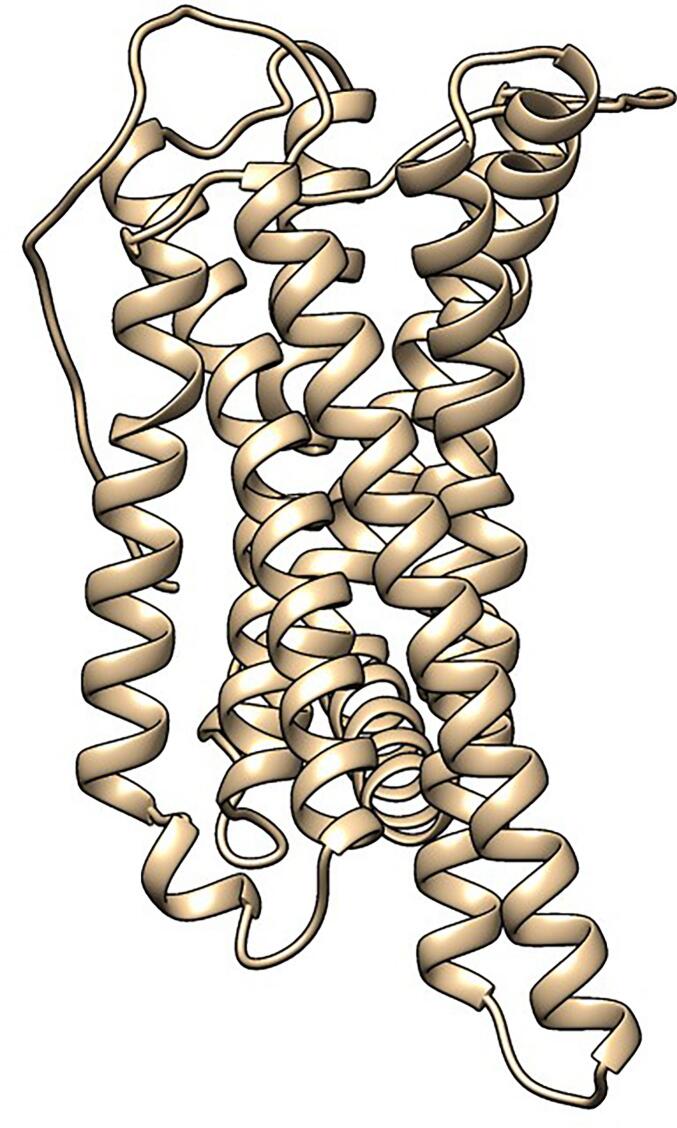
Secondary structure prediction of sheep MC4R protein using SOPMA. The plot shows the distribution of α-helices, β-sheets, turns, and random coils along the protein sequence. The predominance of α-helical regions reflects the typical transmembrane organization of GPCR proteins.

The secondary structure of the sheep MC4R protein, consisting of 332 amino acids, was predicted using the SOPMA tool. The analysis showed that the protein is predominantly composed of α-helices, accounting for 43.67% of the residues (145 amino acids), indicating a strong helical structure commonly associated with transmembrane receptors. Random coils were also prominent, representing 43.37% of the sequence (144 residues). Extended β-strands constituted only 12.95% (43 residues), and no β-turns or β-bridges were predicted (Fig. 3).

### 3.5 Prediction and validation of the sheep MC4R 3D structure

**Fig. 4:**
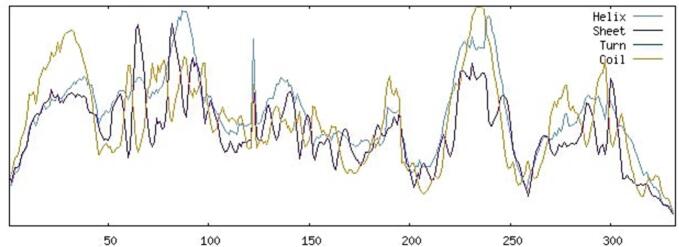
Predicted three-dimensional structure of the wild-type MC4R protein… The structure was generated using I-TASSER and refined with GalaxyWEB. The model shows the characteristic helical architecture of G-protein-coupled receptors, providing a structural framework for mapping nsSNPs and analyzing their potential effects.

The three-dimensional structure of the native sheep melanocortin 4 receptor (MC4R) protein was initially predicted using the I-TASSER server. To enhance the accuracy of the initial model, structural refinement was performed using the GalaxyWEB server (Fig. 4).

**Fig. 5:**
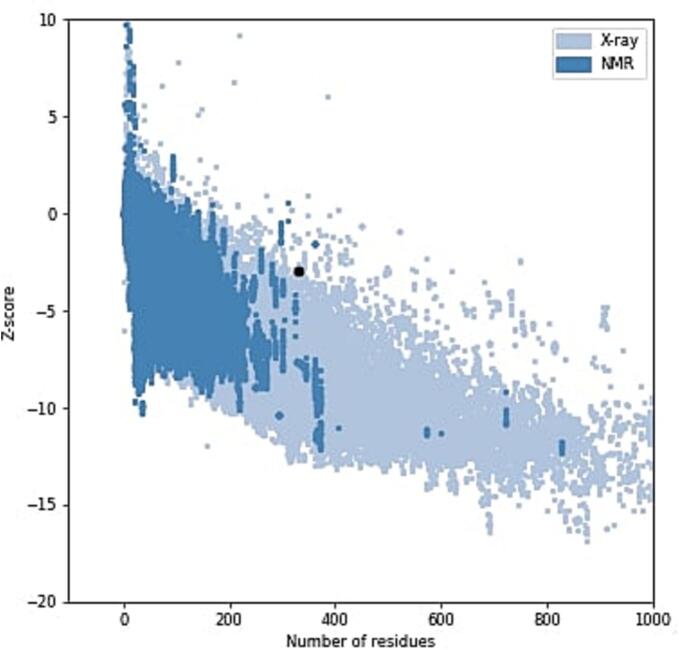
Ramachandran plot analysis of the refined sheep MC4R model generated by PROCHECK. The distribution of backbone dihedral angles (φ and ψ) shows that 92.1% of residues are located in the most favored regions, indicating good stereochemical quality. A small proportion of residues are present in allowed and disallowed regions, which is acceptable for predicted models.

**Fig. 6:**
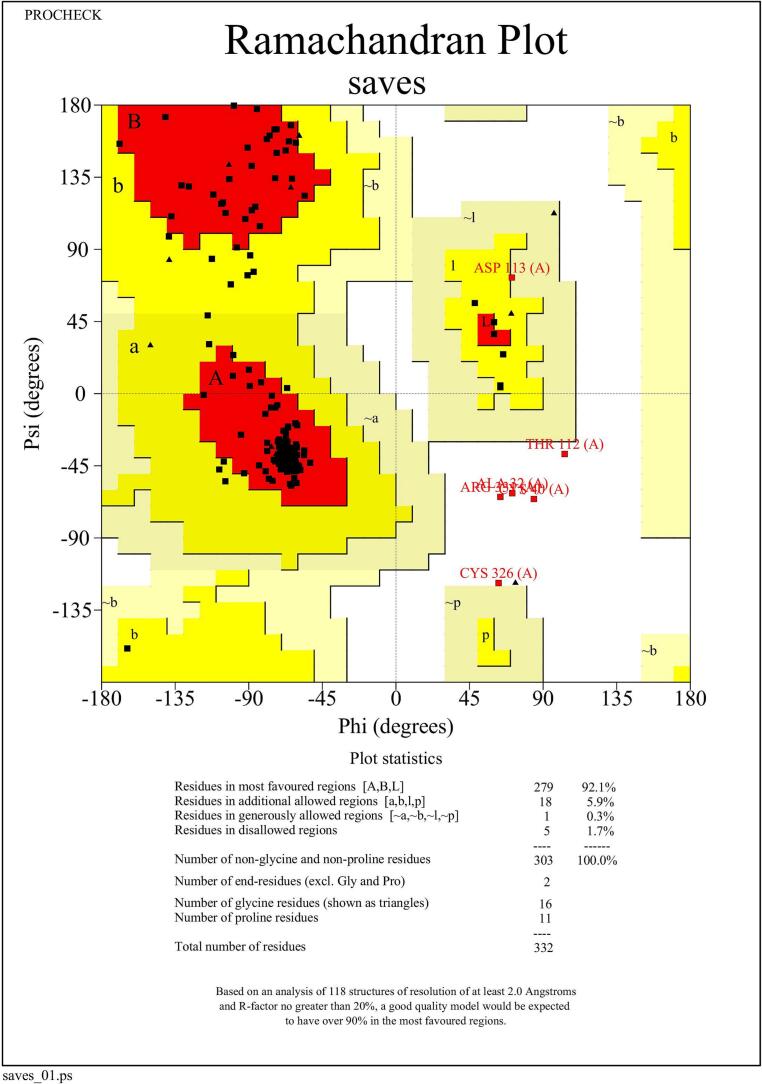
Z-Score validation of the sheep MC4R protein model using ProSA-web. The Z-score indicates that the predicted model falls within the range of native proteins of similar size, supporting its structural reliability.

The refined model was validated using two established tools: PROCHECK and ProSA-web. Ramachandran plot analysis performed by PROCHECK indicated that 92.1% of residues were located in the most favored regions, 5.9% in additionally allowed regions, 0.3% in generously allowed regions, and only 1.7% in disallowed regions (Fig. 5). These values reflect good stereochemical quality for a predicted model. Furthermore, ProSA-web evaluation yielded a Z-score of − 2.98 (Fig. 6), which lies within the range expected for native proteins of comparable size resolved by X-ray crystallography.

### 3.6 Prediction of protein–protein interactions for sheep MC4R

**Fig. 7:**
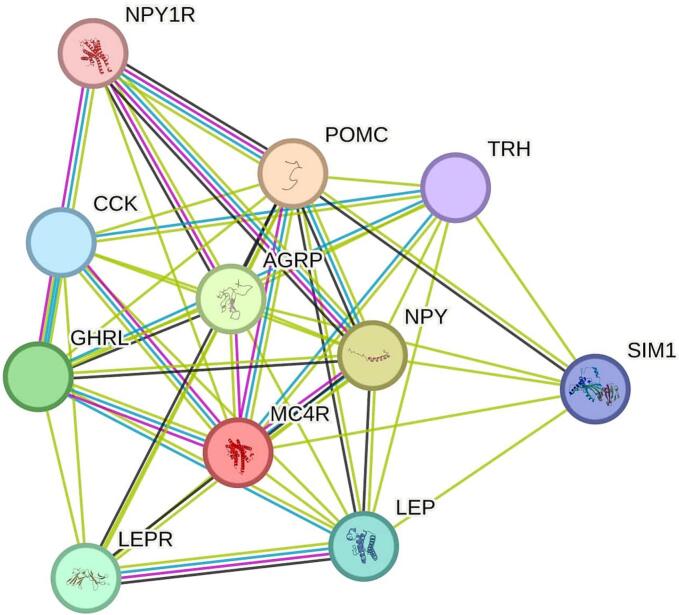
Protein–Protein Interaction network of sheep MC4R and its functional partners predicted by STRING database. Nodes represent proteins, while edges indicate functional associations based on experimental data, co-expression, and computational prediction. MC4R is centrally connected to key regulators of energy balance, including POMC, NPY, AGRP, and LEPR, highlighting its role in metabolic pathways.

Protein–protein interaction (PPI) analysis of the sheep MC4R protein was conducted using the STRING database to identify its functional network and interacting partners. In the resulting interaction map (Fig. 7), each node represents the protein product of a single protein-coding gene. MC4R was found to interact with several high-confidence functional partners, most notably POMC (interaction score: 0.999), which encodes multiple biologically active peptides involved in cortisol release and skin pigmentation. Other strong predicted interactors include NPY (0.973), associated with appetite regulation and gonadotropin hormone secretion; AGRP (0.951), a known antagonist of MC4R; GHRL (0.896) and LEP (0.874), both involved in the regulation of energy balance and food intake; and LEPR (0.875), the leptin receptor. Additional predicted partners, such as CCK (0.861), SIM1 (0.860), TRH (0.805), and NPY1R (0.800), are involved in neuroendocrine signaling, appetite regulation, and metabolic homeostasis.

The PPI network constructed for sheep MC4R consisted of 11 nodes and 46 edges, with an average node degree of 8.36 and a local clustering coefficient of 0.895, indicating a highly interconnected and biologically relevant network. The number of observed interactions significantly exceeded the expected number of 11 edges, with a PPI enrichment p-value of 9.99 × 10⁻^1^⁶, confirming that these proteins are functionally associated rather than randomly linked. This supports the conclusion that MC4R and its interactors participate in a shared biological pathway.

### 3.7 Molecular dynamics simulation analysis

To assess the structural stability, flexibility, and interaction patterns of the system during molecular dynamics simulations, several trajectory-based properties were analyzed, including hydrogen bonding, radius of gyration (Rg), root mean square deviation (RMSD), root mean square fluctuation (RMSF), and solvent accessible surface area (SASA).

**Fig. 8:**
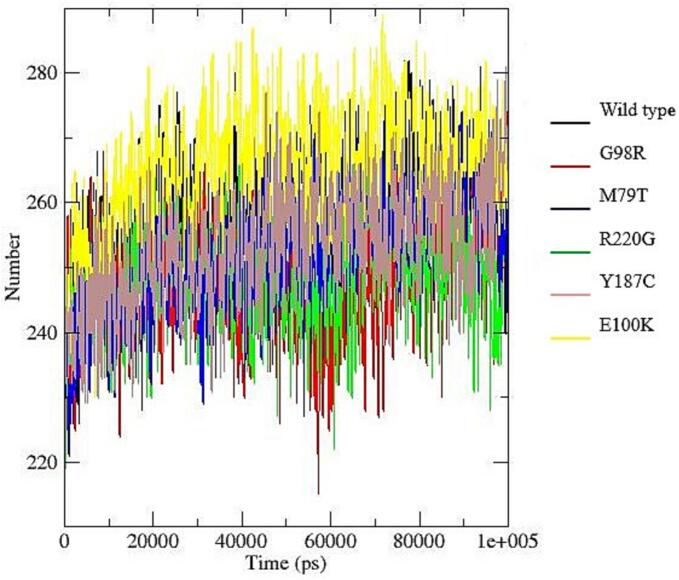

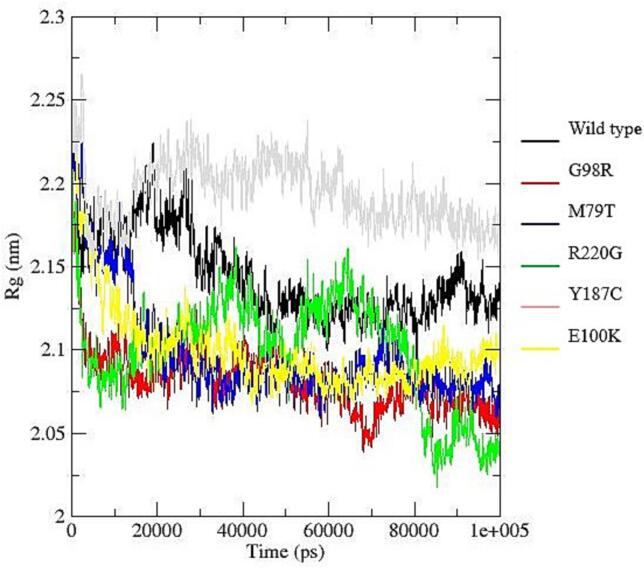

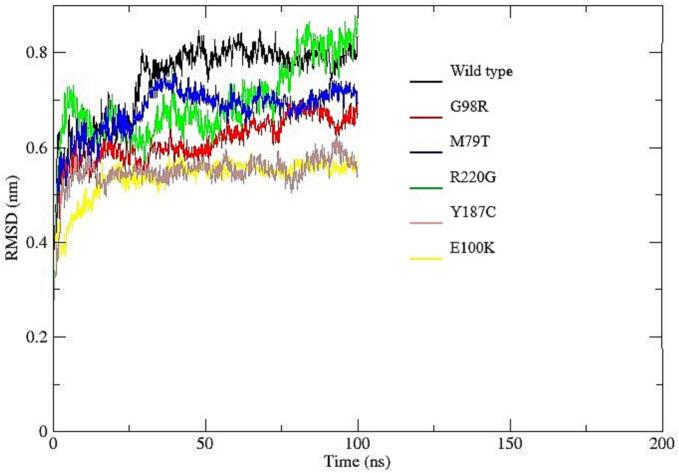

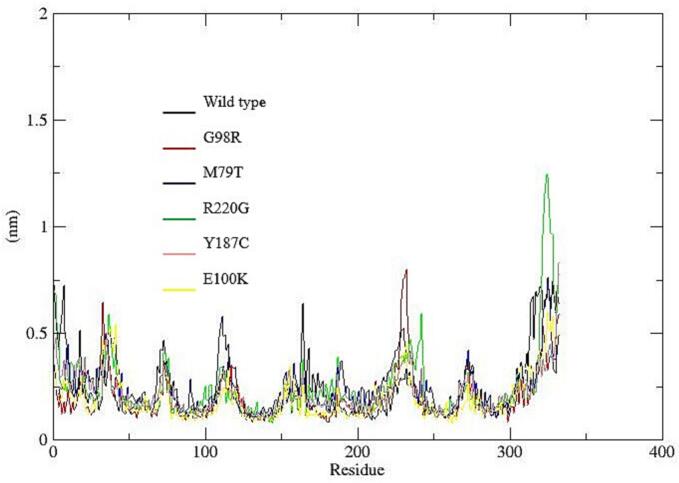

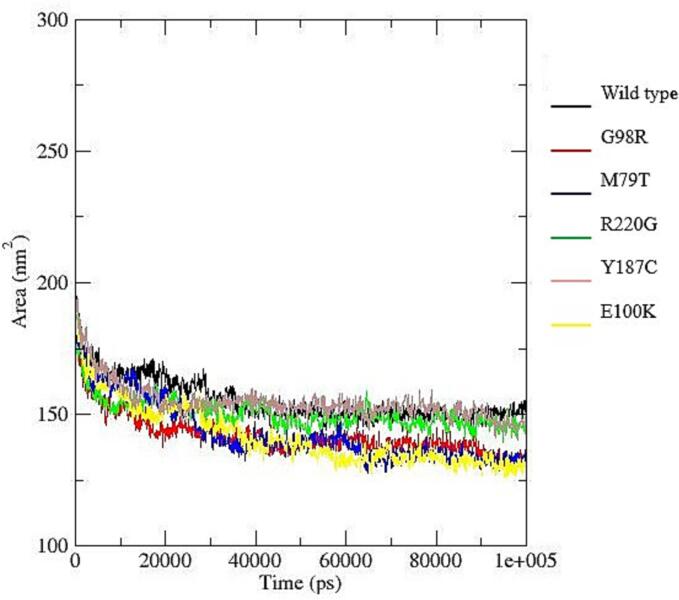
Structural and dynamic properties from molecular dynamics simulations: (a) Time evolution of intramolecular hydrogen bonds showing relatively stable patterns across all systems, with slight variations among mutants indicating differences in internal stabilization; (b) Radius of gyration (Rg) profiles demonstrating overall compactness of the protein structures, where most variants maintain comparable folding behavior, although minor fluctuations suggest mutation-induced structural rearrangements; (c) Root mean square deviation (RMSD) trajectories indicating that all systems reach equilibrium after the initial phase, with certain mutants (e.g., R220G) exhibiting higher deviations, reflecting reduced structural stability compared to the wild type; (d) Root mean square fluctuation (RMSF) per residue revealing localized flexibility differences, particularly in specific regions where mutants show increased fluctuations, suggesting potential functional impacts; (e) Solvent accessible surface area (SASA) variation illustrating differences in protein surface exposure, with some mutants displaying slightly reduced or increased values, indicating altered folding and solvent interaction.

Hydrogen Bond Analysis (Fig. 8a), showed broadly similar trends across the six datasets (wild type–E100K). The average number of hydrogen bonds ranged from 247.97 (G98R) to 265.55 (Y187C). Dataset M79T exhibited the lowest variability, suggesting stable interactions, whereas R220G showed the greatest fluctuations.

The Radius of Gyration (Rg) values (Fig. 8b), ranged from 2.082 nm (G98R) to 2.195 nm (E100K). E100K maintained the highest average Rg with low variability, reflecting a slightly more expanded conformation, while G98R exhibited the lowest average Rg, indicative of a more compact structure.

Root Mean Square Deviation (RMSD) analysis (Fig. 8c), indicated stable structural behavior, with mean values between 0.535 nm (Y187C) and 0.742 nm (wild type). Wild type showed the largest average deviation and variability, suggesting greater conformational drift, whereas Y187C and E100K were the most stable. RMSD values for all datasets remained below 1.0 nm, consistent with the absence of large-scale unfolding events.

Root Mean Square Fluctuation (RMSF) results (Fig. 8d), revealed overall low residue-level fluctuations, with average values ranging from 0.196 nm (Y187C) to 0.251 nm (M79T). M79T displayed the greatest variability, indicating flexible regions, possibly loops or termini, whereas Y187C and G98R showed more rigid profiles. Most residues fluctuated below 0.3 nm, confirming a predominantly stable structure.

Solvent Accessible Surface Area (SASA) values (Fig. 8e), ranged from 141.76 nm^2^ (Y187C) to 155.37 nm^2^ (wild type). Wild type had the highest solvent exposure, while Y187C and G98R were the most compact. R220G and Y187C exhibited the largest variability, reflecting conformational breathing motions, whereas M79T showed the most stable solvent exposure.

## 4 Discussion

The MC4R gene has been extensively investigated across multiple species, where its variants have been associated with key traits such as growth, feed intake, fat deposition, and energy homeostasis. In livestock species, including cattle, pigs, and sheep, polymorphisms in MC4R have been linked to economically important characteristics such as body weight, carcass composition, and growth performance. Several studies have demonstrated that specific amino acid substitutions in MC4R can influence receptor signaling efficiency and ligand binding, thereby affecting metabolic regulation.

Genome-wide studies further support the importance of genetic variation in shaping phenotypic diversity. For instance, Bovo et al., (2020) ^28^ identified more than 22 million SNPs and 359 genomic regions under selection in European pig breeds and wild boars, highlighting the role of selection and introgression in driving genetic diversity and adaptation. Similarly, Hernández-Montiel et al., (2026) ^29^ applied an integrative bioinformatics approach to reveal key biological pathways, including SMAD/TGF-β signaling, underlying economically important traits in pigs.

At the gene-specific level, MC4R polymorphisms have also been associated with production traits in cattle. Huang M, et al (2029) ^30^ identified five SNPs in the coding region of MC4R, with one variant (G1069C) significantly associated with backfat thickness, while strong linkage disequilibrium was observed among several loci. In addition, Maharani et al., (2028) ^31^ reported that the SNP g.1133C > G was associated with increased birth body length, further supporting the role of MC4R variation in growth-related traits.

In this context, the nsSNPs identified in the present study, particularly R220G, M79T, and N294S, may represent functionally relevant positions within conserved regions of the protein. Their predicted destabilizing effects and localization in structurally or evolutionarily important regions suggest a potential impact on receptor function. However, these interpretations should be considered cautiously, as they are based on computational predictions and require further experimental validation.

The present study provides a comprehensive *in silico* evaluation of non-synonymous single nucleotide polymorphisms (nsSNPs) in the ovine *MC4R* gene, revealing their potential impact on protein function, structure, and evolutionary conservation. This work is among the first to systematically assess sheep MC4R nsSNPs using a comprehensive panel of *in silico* tools, including functional prediction algorithms, structural modeling, and protein stability analyses, providing novel integrative insights into the structural and functional consequences of MC4R variants in sheep.

A key strength of the present study lies in its integrative analytical framework, which goes beyond conventional single-tool predictions by combining functional, structural, and dynamic analyses. This approach enables a more reliable identification of high-impact variants and provides mechanistic insights that are often lacking in association-based studies. In the context of sheep genetics, where functional annotations remain relatively limited, such integrative analyses are particularly valuable for prioritizing candidate variants for future experimental validation and incorporation into breeding programs.

Despite the comprehensive computational framework employed in this study, the limitations are acknowledged. The predictions are based entirely on *in silico* tools. The absence of *in vitro* or *in vivo* validation limits the ability to confirm the functional impact of the identified nsSNPs. For future research, experimental validation through site-directed mutagenesis, should be prioritized to confirm the structural destabilization and functional disruption predicted computationally.

Although the integrative computational approach used in this study provides valuable insights into the potential effects of MC4R variants, it is important to interpret these findings with caution. The predictions are based on *in silico* tools that rely on different algorithms and datasets, which may lead to variability in the results. While the use of multiple tools increases confidence in the identified deleterious variants, these predictions do not constitute direct experimental evidence. Therefore, the functional impact of the identified nsSNPs should be considered as potential rather than definitive. Further experimental validation, including *in vitro* functional assays and *in vivo* studies, is required to confirm the biological significance of these nsSNPs.Among the twelve variants analyzed, four nsSNPs (R220G, G98R, E100K, and Y187C) were consistently predicted to be deleterious across multiple functional prediction tools, including SIFT, PolyPhen-2, PhD-SNP, and SNPs&GO. These findings are in line with previous reports suggesting that amino acid substitutions in *MC4R* can affect receptor activity and downstream signaling, influencing traits such as feed intake, fat deposition, and growth in livestock 2, 32.

Protein stability predictions further supported the functional analysis. Variants like M79T, R220G, and G98R showed significant destabilizing effects, with ΔΔG values below –1.0 kcal/mol in I-Mutant and CUPSAT, suggesting that these substitutions may compromise protein folding or disrupt functional conformations. Similar patterns were observed with MUpro and DynaMut, which also highlighted increased flexibility in key regions, particularly for R220G and M79T. Destabilizing nsSNPs are often associated with misfolding or impaired receptor-ligand interactions, which may result in altered signal transduction, as shown in mammalian studies of melanocortin receptors^25^, ^33^.

ConSurf analysis revealed that a majority of the damaging variants were located in highly conserved residues, such as M79T, G98R, and I173M, indicating that these sites are likely essential for structural integrity or function. Amino acid substitutions in conserved regions are more likely to interfere with protein stability or receptor-ligand binding, as supported by previous structural studies of G-protein-coupled receptors (GPCRs) 34, 35.

Secondary structure prediction confirmed the presence of a high proportion of α-helices (43.67%), consistent with the helical transmembrane domains characteristic of GPCRs, including MC4R. The predicted tertiary structure, refined by GalaxyWEB and validated through Ramachandran plot and ProSA-web Z-score (–2.98), confirmed that the model is of good stereochemical quality and suitable for further structural interpretation. The accurate modeling of protein structure is essential for understanding the functional consequences of nsSNPs, particularly when experimental crystal structures are unavailable ^19^.

The protein–protein interaction (PPI) network constructed using STRING highlighted *MC4R* as a central node interacting with key metabolic regulators, such as *POMC*, *NPY*, *AGRP*, and *LEPR*. These interactions are biologically meaningful, given that *MC4R* plays a central role in hypothalamic pathways that regulate energy homeostasis and satiety 1, 23. Variants that alter *MC4R* structure or signaling may therefore have cascading effects on the expression or activity of these interacting partners.

Molecular dynamics (MD) simulations further confirmed the structural instability introduced by deleterious variants such as M79T and R220G. These nsSNPs were associated with increased root-mean-square deviation (RMSD) and flexibility, supporting the predicted loss of stability. Structural fluctuations can impact membrane receptor localization and downstream signaling, as demonstrated in functional assays of MC4R variants in other species 6, 26.

Although the identified nsSNPs showed consistent predictions across multiple computational approaches, their biological significance should be interpreted with caution. In silico analyses provide valuable insights into potential structural and functional effects, but they do not account for population-level variation, gene–environment interactions, or phenotypic outcomes. Therefore, the identified variants should be considered as candidate loci for future investigation rather than definitive markers for selective breeding. Comprehensive validation through population genetics studies, association analyses, and experimental assays will be essential to confirm their practical relevance.

The integration of multiple computational tools provided robust predictions and highlighted variants that warrant further experimental validation. Given the important role of *MC4R* in regulating energy balance and feed efficiency, these findings may provide a basis for future studies exploring marker-assisted selection strategiess in sheep breeding programs, pending further experimental validation. The present study provides a prioritization framework rather than direct evidence for breeding application.

## 5 Conclusion

This study provides a comprehensive *in silico* analysis of twelve non-synonymous single nucleotide polymorphisms in the ovine MC4R gene. Functional predictions identified R220G, G98R, E100K, and Y187C as the most damaging variants. Stability analyses revealed that M79T, R220G, and G98R had the greatest destabilizing effects, with ΔΔG values below –1.0 kcal/mol. Conservation mapping showed that several damaging variants are located in highly conserved regions, indicating their potential structural and functional importance. The refined 3D model demonstrated good stereochemical quality, and protein–protein interaction analysis confirmed MC4R’s central role in energy regulation pathways. Molecular dynamics simulations further supported the destabilizing impact of key nsSNPs, showing increased flexibility and reduced structural stability. These results highlight specific MC4R nsSNPs as candidate variants for further investigation with potential relevance for future selective breeding programs aimed at improving growth and metabolic traits in sheep. Future experimental validation will be essential to confirm these computational predictions and to explore their practical application in livestock genetics.

## 6 Consent for publication

Not applicable.

## 7 Disclosure statement

The authors declare that they have no competing interests. Artificial intelligence (AI) tools (ChatGPT, OpenAI) were used solely to improve the grammar and readability of the manuscript. All scientific content, analyses, interpretations, and conclusions are the original work of the authors.

## CRediT authorship contribution statement

**Anila Hoda:** Writing – original draft, Supervision, Conceptualization. **Sulltane Ajçe:** Writing – review & editing, Resources, Methodology. **Ilia Mikerezi:** Writing – review & editing, Methodology, Investigation. **Xhelil Koleci:** Writing – review & editing, Methodology, Investigation.

## Declaration of competing interest

The authors declare that they have no known competing financial interests or personal relationships that could have appeared to influence the work reported in this paper.
